# General anesthesia, germ cells and the missing heritability of autism: an urgent need for research

**DOI:** 10.1093/eep/dvaa007

**Published:** 2020-07-18

**Authors:** Jill Escher, La Donna Ford

**Keywords:** germ cells, general anesthesia, sevoflurane, autism spectrum disorder, gene expression, DNA damage, halogenated anesthetic gases, intergenerational epigenetic inheritance, transcriptional dysregulation

## Abstract

Agents of general anesthesia (GA) are commonly employed in surgical, dental and diagnostic procedures to effectuate global suppression of the nervous system, but in addition to somatic targets, the subject’s germ cells—from the embryonic primordial stage to the mature gametes—may likewise be exposed. Although GA is generally considered safe for most patients, evidence has accumulated that various compounds, in particular the synthetic volatile anesthetic gases (SVAGs) such as sevoflurane, can exert neurotoxic, genotoxic and epigenotoxic effects, with adverse consequences for cellular and genomic function in both somatic and germline cells. The purpose of this paper is to review the evidence demonstrating that GA, and in particular, SVAGs, may in some circumstances adversely impact the molecular program of germ cells, resulting in brain and behavioral pathology in the progeny born of the exposed cells. Further, we exhort the medical and scientific communities to undertake comprehensive experimental and epidemiological research programs to address this critical gap in risk assessment.

## Introduction

Human gametes and their lineage of precursors are highly specialized cells that undergo stages of complicated molecular programming and meiosis to enable the eventual development of a human, including the unfathomably intricate human brain. Though the germ cells have long been viewed as somewhat passive vessels for transmitting the nucleotide sequence of DNA to the next generation, it has become clear that they also harbor a plethora of molecular factors including structural packaging, transcription factors, histone modifications, chemical groups attaching to DNA and non-coding RNAs that help control how and when genes are expressed over the course of development of the offspring [1–4].

The field of developmental origins of health and disease has expanded beyond early life somatic exposures to also encompass exposures to the germ cells [5]. It is now clear that exogenous toxicants can in some cases induce shifts in the non-genetic machinery in germline, resulting in altered phenotypes in the progeny of the exposed cells [1, 3, 6–10], including impairments in brain and behavior, a phenomenon seen in both mammal and human studies [11, 12]. In experimental models, germ cell exposures to various hormone-disrupting chemicals and drugs have been shown to induce neurobehavioral abnormality in progeny. For example, the fungicide vinclozolin and polychlorinated biphenyls led to socio-sexual behavioral abnormalities in the progeny, especially males, of exposed fetal germ cells in rats [13, 14]. Exogenous or excess hormones also appear to exert an intergenerational impact through fetal germline exposure. For example, betamethasone administered to gestating guinea pigs and rats results in alterations to grandpups’ brain and/or behavior [15–17], and thyroid hormone overexposure in gestating mice leads to abnormal hypothalamic gene expression in grandpups [18]. Nicotine has also been shown to exert intergenerational effects on neurobehavior, with studies in rodents demonstrating that gestating dams’ ingestion of nicotine increased risk for attention deficit hyperactivity disorder (ADHD)-like behaviors in grandpups [19, 20], and similar results with exposure to adult male germ cells [21], with two of these investigations implicating epigenetic mechanisms in the exposed germline [21, 22]. Evidence from rodent models is also emerging that opiate drugs, drugs of abuse and valproic acid could cause neurobehavioral impacts on the next generation via exposed germline [23–27].

Although studies exploring heritable neurodevelopmental impacts of germline toxicant exposure are rare in human cohorts, recent retrospective studies are suggesting this possibility. Grandmaternal pregnancy use of the toxic synthetic estrogen drug diethylstilbestrol (DES) was found to be associated with significantly elevated odds for ADHD in grandchildren, through the exposed female line [28]. Grandmaternal smoking in pregnancy was found to be associated with increased risk for autism traits and diagnosed autism in grandoffspring in the UK’s ALSPAC cohort [29]. And in what is perhaps the most convincing evidence that human germ cell exposures can result in offspring pathology generally, grandmaternal DES has been seen in human studies to increase risks for grandchild pathologies such as urogenital abnormalities and cancers [30].

Although the prevalence of neurodevelopmental abnormality, particularly autism spectrum disorders (ASDs), has increased markedly over the past three decades [31, 32], research programs have largely hunted for etiological clues in the DNA nucleotide sequence or developmental somatic exposures, reflective of the predominant ‘genes or environment’ paradigm. Scant attention has been paid to questions of genetic toxicology, i.e. the potential for exogenously induced, germline-mediated sources of the pathologies [33]. Although many toxicants warrant concern regarding potential heritable effects [33–37], this commentary focuses on one class of pharmaceuticals that we believe deserves urgent attention, general anesthesia (GA), and in particular, the halogenated synthetic volatile anesthetic gases (SVAGs).

From our perspectives as autism and research advocates, several circumstances conspired to raise this concern: autism family histories informally gathered by author J.E. that prompted the initial hypothesis; biological plausibility based on molecular actions and animal research; and the remarkable ability of this concept to reconcile a variety of neurobiological and epidemiological patterns seen in autism research.

Because this genetic toxicology-based hypothesis falls far afield from mainstream approaches to autism etiology, we would like to share some of the autism family histories shared with J.E. that had, quite unexpectedly, suggested to us the potential risk to germ cells posed by a parent’s early life and/or intensive exposure to surgery. Parents of children with idiopathic autism had reported, for example, their own early childhood surgeries for hernia repair, heart defect, cleft lip, burst appendix, adenoid removal and severe burns. A handful of cases involved *in utero* exposure of the autism parent, such as a mother of two girls with autism whose mother had an appendectomy when 7 months pregnant with her. In about half of these cases, exposed parents had more than one child with idiopathic autism. Two stories involved extremely severe idiopathic autism in sons of men who had undergone several successive, complicated surgeries in adolescence or early adulthood following accidental gunshot wounds, and another involved a father who had undergone six surgeries under GA over the course of 8 years, before he fathered two boys with autism. As a rough control group, in all cases where such information was available, the parent’s siblings who did not have similar exposures were said to have typically developing children. In addition, in nearly all cases no other known risk factors for autism were present.

One of us (L.D.F.) is a former anesthesiologist who is familiar with the clinical use of GA compounds in children and adults, and their toxicities and mechanisms of action. Through her many years of training and practice, she saw no concerns raised about potential germ cell impacts to anesthetized patients. Now, familiar with red-flag patterns in autism families and the biological plausibility suggested by the relevant literature, she has grown concerned about the gap in risk assessment, which currently ignores the types of harms that could result from germ cell exposure to GA.

To be clear about the scope of our proposal, we believe that GA must be mostly benign in terms of molecular heritable content within human germ cells; otherwise, considering the magnitude of exposure across the population, we would see pervasive heritable disease and dysfunction in offspring at rates much higher than exist today. Nevertheless, we here consider narrowed possibilities: whether certain GA compounds, in certain developmental windows, and in high single or successive doses may raise the risk for damage to or mis-programming of neurodevelopment-related genes in germ cells, thereby increasing risk for abnormal neurobehavioral phenotypes in offspring.

To probe these questions, we begin with a general discussion about the prevalence, practice and properties of GA, an exposure with which the environmental epigenetics community may lack familiarity. We then turn to the mammalian research finding heritable neurodevelopmental consequences of germline exposure to GA.

## GA and SVAGs in Historical and Clinical Context

Anesthetic agents have been utilized in medicine for millennia, but in the late 1950s, chemists revolutionized the field with the creation of the first of the halogenated SVAGs that were more efficacious in keeping patients sedated and inert during surgical procedures, with reduced risks such as hepatotoxicity and flammability. Halothane was introduced in the late 1950s, methoxyflurane and enflurane in the 1960s, isoflurane in 1972, desflurane in 1992 and sevoflurane, currently the most widely used SVAG in the USA, in 1994 [38]. The gases, though nearly miraculous in enabling modern surgery, also have had their downsides, among them possible lasting neurological damage, particularly to the young patient, resulting in persistent learning and memory deficits [39–42].

SVAGs are small, potent lipophilic molecules that, on an organismal level, are essentially lethal in their physiologially relevant doses: patients, whose brain and muscle functions shut down in a temporaryinduced coma, are typically kept alive only through a breathing apparatus and careful monitoring. The gases diffuse through the body, including vessel-rich tissues, such as the gonads, with the clinical purpose to interrupt nerve signals [43]. The molecules enter the nucleus, altering activity of receptors, signaling processes, chromatin and even DNA, as will be discussed below. To our knowledge, GA represents the most acute neurotoxic exposure most Americans will encounter in their lifetimes.

Although one may be tempted to assume that neonates and young children require lower concentrations of GA due to their small size and immature neurology, the opposite is often true. GA gases are used in higher concentrations in infants under the age of 1 year, and also for young children owing to the different pharmacokinetics and gamma-amino butyric acid (GABA)-ergic function in the young child. Notably, the Type A GABA (GABAA) receptor, the main target of sevoflurane, is excitatory rather than inhibitory in the immature neurons [44]. Interestingly, the new field of antenatal corrective surgery requires particularly high concentrations of sevoflurane to relax the uterus over many hours, exposing an undeveloped fetus to unprecedented quantities of GA [45]. That said, any pregnancy GA exposure generally will reach the embryo or fetus, exposing the developing germline, an important concern since early stage germ cells possess heightened vulnerabilities to toxicants [30].

GA has become an increasingly prevalent exposure, used primarily for surgeries, but also for diagnostics, imaging and some dental procedures. Today, 6 million children (1.5 million of whom are infants) and up to 75 000 pregnant women undergo inpatient surgical procedures each year [39]. Overall, in 2010, 51.4 million inpatient procedures were performed in non-federal hospitals in the USA [46], and 48.3 million surgical and non-surgical procedures were performed during 28.6 million ambulatory surgery visits to hospitals and ambulatory surgery centers combined [47]. One study based on procedures in three states in 2002, found Americans undergo an average of 9.2 surgical procedures (3.4 inpatient operations, 2.6 outpatient operations and 3.2 non-operating room invasive procedures) per lifetime [48].

Although experimental research is often conducted on a single GA agent, it is important to keep in mind that in reality, surgical procedures typically involve multiple anesthetic medications, including not just inhalation anesthesia but also intravenous agents such as propofol and fentanyl, benzodiazepenes, regional anesthetics and other drugs like muscle relaxants. The combination and doses of drugs used will depend on the patient characteristics, demands of the particular procedure, cost and availability of medications and habits and judgment of the practitioner. Combinations of agents may be significantly more neurotoxic than anesthetics given in isolation, as was shown with isoflurane administered in combination with nitrous oxide and midazolam [49]. Because of the need for preparation, in addition to recovery, even relatively minor surgeries can require an hour or more of GA: a study from Brazil found the overall mean duration of anesthesia among all operations across multiple specialties was nearly 3 h: 178.12 ± 110.46 min [50].

In addition, the circumstances around many surgeries and medical procedures involve other toxic elements which could independently exert germ cell impacts or exacerbate GA effects. For example, high concentrations of oxygen may be utilized in neonatal procedures, which could induce oxidative stress [51]. Because volatile anesthetics have been shown to damage mitochondria, oxidative stress can amplify injury to mitochondria [52, 53]. Further, a patient may require several successive surgeries, resulting in overall exposure that could intensify molecular damage, or limit opportunities for DNA repair. In addition to the drugs administered to induce anesthesia, patients may experience high levels of endogenous stress hormones, infectious microbes or other medications, including potent analgesics and steroid drugs, related to their trauma, condition or injury.

## Cellular, Genetic and Epigenetic Toxicities of SVAGs

The ultimate biological effect of GA exposure on germ cells, including potential heritable impacts, depends on many processes within the germ cell. An increase in DNA damage, combined with failure of repair, could result in *de novo* germline mutagenesis, and therefore heritable disease. Similarly, damage to the transcriptional or epigenetic machinery in the germ cells could lead to impaired gene expression in the offspring, even absent alterations of the nucleotide sequence.

Although SVAGs are not known as somatic mutagens and are not classified as carcinogens [54], it is known that the gases can cause significant increases in DNA damage in exposed somatic cells [55, 56]. This phenomenon has been observed in studies of tissues both at the site of contact, e.g. epithelial cells in the nose and bronchoalveolar cells [57, 58] and systemically, e.g. peripheral blood lymphocytes (PBLs) [56, 59–64] although those risks seem to be agent and dose/duration dependent [56, 65]. GA waste gases are even seen to induce DNA damage in operating room personnel, with chronic exposure inducing cumulative genotoxic effects [66, 67].

We could locate just a single study of DNA damage in germ cells caused by these chemicals, and it was performed on human sperm in vitro [65]. In this study, genotoxic activities of different concentrations of halothane, isoflurane, sevoflurane and desflurane were investigated in both human PBLs and sperm cells by alkaline comet assay. The comet assay is single-cell gel electrophoresis that measures DNA strand breaks as well as alkali-labile sites by tail intensity. The study showed that all analyzed gases were capable of inducing DNA damage on PBLs in a dose-dependent manner. However, the results in sperm were somewhat different. Although isoflurane and sevoflurane caused dose-dependent damage in sperm, genotoxic effects of desflurane were not observed and the genotoxic effect of halothane was not dose dependent.

The existence of DNA damage to sperm is perhaps not surprising considering that beginning nearly four decades ago, with Land *et al*. [68], there has been a steady stream of research observing that SVAGs can damage the morphology, integrity and concentration of mammalian and human male germ cells [69–75]. As with most aspects of germ cell toxicology, information about the fate of oocytes is rare compared with the male gametes, but studies in rodents suggest isoflurane and sevoflurane reduce oocyte quantity and quality [76, 77]. SVAGs are also widely observed to act as steroid hormone disruptors, a chemical force observed to induce dysfunction in the gonadal tissues and cells [69, 70, 73–75, 77]. It is also worth noting that the hormone-disrupting properties of SVAGs should also raise concerns regarding potential heritable effects, considering the well-documented phenomenon of generational impact of endocrine-disrupting chemicals [13, 78].

Beyond the molecular phenomenon of DNA damage, SVAGs also induce epigenetic and chromatin modifications that alter neural gene expression [39, 79–89]. In the studies, faulty gene expression was seen to lead to a variety of defects of neural development and function, including errors of protein synthesis, cytoskeleton formation, signal transduction, synaptic plasticity, dendritic spine density and levels of the brain-derived neurotrophic factor (BDNF), among other defects. In one recent study in rats, exposure to sevoflurane triggered a cascade of molecular events including a more condensed chromatin structure less conducive to transcription of the target genes *BDNF* and *c-Fos*, which are critical for cognitive development. The impairment in proper dendritic arborization led to impaired neuronal connectivity resulting in faulty formation of neuronal circuits and compromised synaptic neurotransmission [82]. Another recent study in rats found that isoflurane led to diminished expression of proteins critical for neuronal migration, resulting in incorrect cortical positioning [39].

In summary, we can see that SVAGs can cause DNA damage in somatic cells, with emerging evidence for the same in human sperm cells. It has also become apparent that the gases can induce non-genetic damage in neural cells, inducing dysregulation of neuronal development and function. For us, however, the question must go a step further—can GA damage genetic or non-genetic heritable elements in germ cells in such a way to raise the risk for pathology in offspring, specifically neurobehavioral impairment? To date, the five published mammalian studies looking at this question have yielded affirmative answers, and we will discuss them in some detail.

## Germline Exposure to GA Raises Risk for Offspring Brain and Behavior Pathology

The five studies conducted to date in mammalian models to investigate whether germline exposure to GA could exert adverse phenotypic outcomes in live-born progeny all involve SVAGs. The first two come from the early 1980s from the laboratory of Herman Turndorf, MD, Professor and Chairman of Anesthesiology at the New York University School of Medicine. Turndorf and his colleagues looked at the effects halothane and enflurane had on the learning and behavior of mice exposed in the womb. In a succinct 1981 paper, the team reported that the offspring born of gestating females exposed to halothane and enflurane suffered long-term learning impairment, performing poorly in food maze tests compared with control mice [90]. But the researchers also did something rather remarkable for their time in that they also examined learning outcomes in six mice in the following generation, i.e. the mice which were in essence early stage eggs nested inside fetal ovaries of their fetal mothers at the time of exposure. The researchers found these grandpups to be ‘significantly slower than control mice throughout the training’ on all days of testing and all configurations of the maze test. They concluded that the grandpups’ impaired learning ‘suggests that the anesthetic agent may have caused a genetic aberration’ in the exposed mothers’ fetal eggs.

After this article, the lab returned to the question of germ cell effects of GA in a different type of mouse experiment. Knowing that enflurane induced morphologic abnormality in murine sperm (citing Land *et al*. [68]) and that halothane caused learning impairments in the generation born of exposed eggs based on their own observations, they investigated the possibility that exposure of adult male mice to enflurane prior to mating could also affect the brain function of offspring, due to damage to the exposed sperm. Once again, they found impaired learning function in the generation born of the exposed germ cells, this time later-stage sperm instead of early stage eggs. They remarked that it ‘seems likely that spermatogenetic changes, caused by enflurane, are associated with genetic alterations’ that affected the pups’ brain development [91].

After this pair of studies raising the spectre of potential mental impairment in progeny via ‘genetic aberrations’ or ‘genetic alterations’ of female or male germ cells, the question of potential heritable impacts of GA seemed to fall into the abyss, with no papers published on this topic for more than three decades. Though it is difficult to say why the concept seemed to vanish, it seems possible that the observations reported by Turndorf’s lab fell victim to the weight of conventional dogma about inheritance. The GA agents were not thought to be mutation-causing, so the idea that GA could induce a heritable neurobehavioral pathology possibly amounted to a sort of scientific heresy.

However, recent years have seen a renewed attention to this concept. Anatoly Martynyuk, PhD, Professor of Anesthesiology and Neuroscience at the University of Florida, hypothesized that sevoflurane, by acting as a stressor and endocrine disruptor, in addition to affecting brain development in the directly exposed neonatal rats, could epigenetically reprogram their germ cells and, therefore affect brain development in their future offspring. To test these hypotheses, Martynyuk’s lab exposed both male and female neonate rat pups to sevoflurane and then looked at brain, gene expression and behavior in the exposed parents and in offspring of all combinations of exposed and control sires and dams [92].

The lab used a sub-surgical dose of sevoflurane because a surgery sufficient dose would have resulted in low oxygen levels and other abnormalities in the pups’ blood (use of GA generally requires use of a breathing apparatus to keep the patient alive, something the researchers could not do in 5-day-old rats). They looked at the expression of the KCC2 gene known to be essential for proper age-dependent functioning of GABA Type A receptors (GABA_A_R) signaling. GABA_A_R can be excitatory and inhibitory in early postnatal brain and adult brain, respectively. The age-dependent transition in GABA_A_R signaling from excitatory to inhibitory is mainly due to developmental increases in expression of KCC2. Impairments in age-dependent increases in KCC2 may be a risk factor for the emergence of neurodevelopmental disorders [93]. After assessing the effects in the neonatally exposed to sevoflurane sires and dams (which suffered neuroendocrine abnormalities, including impaired expression of hypothalamic and hippocampal KCC2) the team looked at brain, gene expression and behavior outcomes in the generation born of the pups’ GA-exposed germ cells.

They found that the male, but not female, progeny showed signs of neurodevelopmental impairment. Progeny of exposed males, that is, of the exposed sperm, had abnormalities in the maze test, suggesting increased anxiety-type behavior, abnormalities in prepulse inhibition of startle, suggesting decreased ability to filter out unnecessary information and decreased expression of the KCC2 gene in the hypothalamus. Where both parents were exposed to the sevoflurane as pups, male progeny exhibited impaired spatial memory and decreased expression of the gene in both the hypothalamus and hippocampus. An analysis of epigenetic changes in sperm of exposed males and brains of progeny revealed KCC2 gene expression shifts not present in control rats. In other words, it appeared that the male rat progeny, exposed only during the early germ cell stage, exhibited behavioral impairments connected to sevoflurane-induced epigenetic modification [92].

These findings suggest that sevoflurane could induce a non-genetic effect in early stage germ cells, causing some sex-specific brain and behavioral abnormality in the next generation, even when used at low concentrations. A *British Journal of Anaesthesia* editorial accompanying Martynyuk’s paper, and also citing the first Turndorf study, touched on the possible public health implications of the new findings. The commentary, ‘A poisoned chalice: the heritage of parental anaesthesia exposure,’ noted that ‘we are faced with a real possibility that general anaesthetics are not innocuous agents that “only put children to sleep” but rather formidable modulators of chromatin remodeling and function’ perhaps modulating developmental neuroplasticity in the next generation [83].

As germ cells are susceptible to reprogramming by environmental factors across the lifespan, Martynyuk’s lab also tested whether sevoflurane could induce neurobehavioral abnormalities in offspring when the anesthetic is administered to young adult rats [94]. They found similar, not identical, abnormalities in parental germ cells and in male offspring of exposed sires and dams. The researchers concluded that sevoflurane exposure affects brain development in male offspring by epigenetically reprograming both parental germ cells, when administered to parents over a broad age range, from neonates to young adults.

The lab of Vesna Jevtovic-Todorovic, PhD, MBA at University of Colorado recently performed an experiment with some similar aims as Martynyuk’s, but looking only at the offspring brain as an endpoint, not the animals’ behaviors. After exposing neonatal female rats to 6 h of sevoflurane, they bred the females, and found their offspring’s brains exhibited epigenetic abnormalities, including reduced DNA methylation, an effect linked to functional decline in learning and memory. An upregulation of Arc and JunB mRNA expression, 71.6% and 74.0%, was seen in the male offspring. Also hypomethylation and modifications to immediate early genes crucial to normal neuronal morphological development and synaptic plasticity were observed. The alterations in the offspring brain suggested to the researchers that sevoflurane causes epigenetic modifications in the early rat oocytes [95].

Taken together, we believe that these five studies provide an important proof of principle that SVAGs can enter the germ cell nucleus, alter chromatin and epigenetic programming, change transcription of key brain development genes and induce adverse neurodevelopmental outcomes in progeny, particularly males. Although they are too preliminary to justify any conclusions regarding possible impacts in humans, they at a minimum suggest that this toxicological phenomenon exists and warrants study in humans.

Unfortunately, to our knowledge no such studies have yet been conducted in humans, either prospectively or retrospectively. Although there is some, if insufficient, regulatory concern for pharmaceutically induced germ cell mutagesis [96], potential germline modification via other mechanisms, for any dose, combination or concentration, and during any window of exposure, is not the target of US Food and Drug Administration regulatory effort or risk assessment [33], and GA is no exception.

Based on this research, it seems that while the DNA damaging properties of SVAGs could possibly raise the risk for *de novo* germline mutation where there is failure of DNA repair, epigenetic and transcriptional mechanisms may be more commonly at work. Whether genetic or non-genetic, however, the ultimate question for human health is the same: the possibility for heritable pathology, including neurodevelopmental disruptions in offspring.

## Rethinking Genetics as the Sole Source of Heritability in ASDs

Over the past decade, the strong heritability of autism has moved the field of autism etiology research strongly toward questions of genetic causation, particularly the search for rare and common variations in the nucleotide sequence of affected individuals [97]. This line of research, however, has largely failed to uncover the roots of the so-called ‘missing heritability’ of autism.

Without question, autism is a strongly heritable neuropsychiatric disorder—if an older sibling has autism, the risk of recurrence in a younger sibling is sharply higher than in unaffected families [97–99]. Heritability models applied to population-wide cohorts have pegged autism’s heritability at around 80% [100–102], with scant evidence for somatic-level influences, sometimes called maternal or environmental influences.

Yet, this heritability is idiosyncratic and generation-specific, as the heightened risk has been found only among siblings (and to a lesser extent half-siblings and cousins), and not parent to child, grandparent to child or otherwise up an ancestral tree. To our knowledge, no studies in genetics or populations have demonstrated that autism, or the substantially disabling neurodevelopmental pathologies we now call autism, travels ancestrally down the generations to any significant extent. In fact, the largest genetic contributor to autism risk identified so far is not inherited genes but instead *de novo* mutations in parental gametes, which may explain about 10% of overall cases [97, 103].

Locating the missing heritability of autism has been the focus of immense effort among genetics researchers, many of whom have contended that the portion of heritable risk not explained by rare pathogenic mutation must rest in common and normally harmless single-nucleotide polymorphisms acting additively. This interest seems to be rooted in papers such as Gaugler *et al*. [104] and Klei *et al*. [105], which are frequently cited to suggest that most of the risk for autism is caused by common variation. However, we are puzzled by the biological assumptions feeding these statistical models, which equated heritability to genetics and presumed exogenous factors could act only at the somatic, not germline, level. The models and their interpretations have recently been subject to criticism owing to their over-simplicity and faulty logic [106]. Furthermore, a recent robust attempt to actually locate common genetic risk variants for autism, while identifying five genome-wide-significant loci, could not account for more than an insignificant fraction of the overall population risk [107]. Further, a recent review and re-evaluation of genome-wide association studies found that almost no of autism risk could be predicted accurately from single-nucleotide polymorphisms, and the authors cautioned that while twin studies tend to find strong heritability, heritability may be over-estimated due to unexamined epigenetic effects [108].

To capture the true complexities likely underlying autism etiology, we suggest an end to the false dichotomy of ‘genes or environment’ that has long driven directions in autism academia, dividing the field into two flanks: one focused on the DNA sequence, and the other eyeing prenatal and other somatic stressors. Instead, we suggest the field should adopt a more biologically authentic view of heritability that includes toxicant exposure, germ cell mutagenesis and epigenetic/chromatin perturbations that can disrupt gene expression in offspring neurons. Instead of a ‘genetics first’ approach to autism research, which emphasizes a hunt for variations absent any concern for exposure context [109], we suggest a revision to a ‘genetic toxicology first’ approach, which incorporates parent exposure histories as a critical variable in understanding heritability and pathogenesis in offspring.

As autism rates continue to climb, now reaching an estimated 1 in 54 US children according to the Centers for Disease Control [31], the dominance of over-simplistic assumptions about molecular heritability is thwarting desperately needed progress, and based on the literature and our own observations in the community we can think of no other germ cell exposure that deserves more attention than GA, owing to its potent ability to disrupt the proper expression of genes important for early brain development. This work is by nature interdisciplinary, and we echo the words of renowned genetic toxicologists who recently stated, ‘collaborative work between clinicians, epidemiologists, genetic toxicologists, genomics experts and bioinformaticians [is needed] to precisely define how environmental exposures impact germ cell genomes’ [96].

## Heritable Impacts of GA May Help Explain Findings in Autism Research

Although we hardly hypothesize that heritable impacts of GA are the sole or even largest driver of the sharp increase in the prevalence of ASDs over the past several decades, as it is likely that many factors contribute to autism risk across the population, it is worth noting how this particular hypothesis aligns with findings from autism research, lending to its plausibility.

Notably, it harmonizes with findings from neurobiology. The neuronal defects induced by developmental GA exposure, such as abnormal migration of cortical neurons and impaired synaptogenesis mirror the impairments seen in post-mortem autism brains [110, 111], raising questions about dysregulation of the associated genes via the autistic subject’s germline. It stands to reason, and indeed has been demonstrated in preliminary rodent studies discussed above, that neurodevelopment genes targeted by SVAGs would also be targeted in germ cells. Unlike differentiating neurons, a lesion at the blueprint germ cell level could potentially lead to more acute phenotypic consequences due to systemic interference with the precise tempo-spatial processes of brain development. Because ‘epigenetic dysfunction is a fundamental contributor to brain development and disease pathogenesis of neurodevelopmental disorders, including ASD’ [112], SVAG’s dysregulating effects on brain development genes in germline should raise urgent questions.

The hypothesis is also consistent with observations regarding autism heterogeneity and the ‘broader autism phenotype’ among offspring. GA exposure to male or female germ cells over different times, in different doses, in different combinations, against a backdrop of varying additional exposures and genomic susceptibilities, would likely not cause uniform effects. This roulette-wheel mix could therefore help explain the heterogeneity of the autisms and ‘broader autism phenotype’ in the population more broadly [113]. It may also help explain why parental age is associated with offspring autism risk, even when genetic and somatic environmental factors cannot account for the risk [114], as higher rates of toxicant exposure experienced by the parents over the pre-conception lives can increase risk for germline damage [115].

The hypothesis also has the unusual property of having potential bearing on one of the most puzzling yet consistent epidemiological findings in autism, the skewed sex ratio of approximately four males for each female affected [31]. This phenomenon is consistent with the increased risk for male offspring pathology in intergenerational responses to GA exposure as detected in Ju *et al*. [92, 94]. Additionally, other studies in chemical disruption of germ cells have found male offspring more likely suffer adverse neurobehavioral effects [13].

## Conclusion: Heritable Impacts of GA Should Be a Priority for Toxicology and Autism Research

Modern GA is by all accounts one of the great medical advances of our age. At the same time, we should be cognizant that these agents are powerful poisons that may invite unintended consequences, not just for ourselves, but for our offspring, as these agents can interfere with how the genome gets folded, decorated and expressed, interfering with the proper function of brain development genes. Given the potentially significant public health implications, and the emerging science demonstrating biological plausibility, we urge public and private funders of autism, neurodevelopment and anesthesia research to initiate comprehensive research programs in experimental models and human cohorts. Studies should look at both male and female germline exposures of various intensities and agents, across the span of germ cell developmental windows. The windows should include, at a minimum: embryonic and fetal germline; neonatal and early childhood; adolescence; and pre-conception spermatogenesis.

As we witness a baffling tsunami of young people suffering from serious, permanent functional and behavior impairments—disorders shown to be heritable but not strongly genetic in any classic sense—the public deserves a research program that considers whether some of the causes of this growing crisis may lie in parental germ cells perturbed by powerful genotoxicants like modern GA.

*Jill Escher is an autism research philanthropist, advocate, former lawyer, and mother of two children with nonverbal autism. Learn more about the work of the Escher Fund for Autism at GermlineExposures.org. La Donna Ford, MD, is a retired anesthesiologist and the mother of a son with autism. Both authors are based in the San Francisco Bay Area. This commentary was adapted from a talk delivered by Ms Escher at the Epigenetic Inheritance: Impact for Biology and Society conference in Zürich on 26 August 2019.*
